# Islet Autoantibody Level Distribution in Type 1 Diabetes and Their Association With Genetic and Clinical Characteristics

**DOI:** 10.1210/clinem/dgac507

**Published:** 2022-09-08

**Authors:** Sian Louise Grace, Jack Bowden, Helen C Walkey, Akaal Kaur, Shivani Misra, Beverley M Shields, Trevelyan J McKinley, Nick S Oliver, Timothy J McDonald, Desmond G Johnston, Angus G Jones, Kashyap A Patel

**Affiliations:** Institute of Biomedical & Clinical Science, University of Exeter Medical School, Exeter, Devon EX2 5DW, UK; Institute of Biomedical & Clinical Science, University of Exeter Medical School, Exeter, Devon EX2 5DW, UK; Division of Diabetes, Endocrinology and Metabolism, Imperial College London, London SW7 2AZ, UK; Division of Diabetes, Endocrinology and Metabolism, Imperial College London, London SW7 2AZ, UK; Division of Diabetes, Endocrinology and Metabolism, Imperial College London, London SW7 2AZ, UK; Institute of Biomedical & Clinical Science, University of Exeter Medical School, Exeter, Devon EX2 5DW, UK; Institute of Biomedical & Clinical Science, University of Exeter Medical School, Exeter, Devon EX2 5DW, UK; Division of Diabetes, Endocrinology and Metabolism, Imperial College London, London SW7 2AZ, UK; Institute of Biomedical & Clinical Science, University of Exeter Medical School, Exeter, Devon EX2 5DW, UK; Academic Department of Clinical Biochemistry, Royal Devon and Exeter NHS Foundation Trust, Exeter, Devon EX2 5DW, UK; Division of Diabetes, Endocrinology and Metabolism, Imperial College London, London SW7 2AZ, UK; Institute of Biomedical & Clinical Science, University of Exeter Medical School, Exeter, Devon EX2 5DW, UK; Macleod Diabetes and Endocrine Centre, Royal Devon and Exeter NHS Foundation Trust, Exeter, Devon EX2 5DW, UK; Institute of Biomedical & Clinical Science, University of Exeter Medical School, Exeter, Devon EX2 5DW, UK; Macleod Diabetes and Endocrine Centre, Royal Devon and Exeter NHS Foundation Trust, Exeter, Devon EX2 5DW, UK

**Keywords:** type 1 diabetes, glutamate decarboxylase autoantibodies (GADA), islet antigen-2 autoantibodies (IA-2A) and zinc transporter 8 autoantibodies (ZnT8A)

## Abstract

**Context:**

The importance of the autoantibody level at diagnosis of type 1 diabetes (T1D) is not clear.

**Objective:**

We aimed to assess the association of glutamate decarboxylase (GADA), islet antigen-2 (IA-2A), and zinc transporter 8 (ZnT8A) autoantibody levels with clinical and genetic characteristics at diagnosis of T1D.

**Methods:**

We conducted a prospective, cross-sectional study. GADA, IA-2A, and ZnT8A were measured in 1644 individuals with T1D at diagnosis using radiobinding assays. Associations between autoantibody levels and the clinical and genetic characteristics for individuals were assessed in those positive for these autoantibodies. We performed replication in an independent cohort of 449 people with T1D.

**Results:**

GADA and IA-2A levels exhibited a bimodal distribution at diagnosis. High GADA level was associated with older age at diagnosis (median 27 years vs 19 years, *P* = 9 × 10^−17^), female sex (52% vs 37%, *P* = 1 × 10^−8^), other autoimmune diseases (13% vs 6%, *P* = 3 × 10^−6^), and *HLA-DR3-DQ2* (58% vs 51%, *P* = .006). High IA-2A level was associated with younger age of diagnosis (median 17 years vs 23 years, *P* = 3 × 10^−7^), *HLA-DR4-DQ8* (66% vs 50%, *P* = 1 × 10^−6^), and ZnT8A positivity (77% vs 52%, *P* = 1 × 10^−15^). We replicated our findings in an independent cohort of 449 people with T1D where autoantibodies were measured using enzyme-linked immunosorbent assays.

**Conclusion:**

Islet autoantibody levels provide additional information over positivity in T1D at diagnosis. Bimodality of GADA and IA-2A autoantibody levels highlights the novel aspect of heterogeneity of T1D. This may have implications for T1D prediction, treatment, and pathogenesis.

Islet autoantibodies are commonly used in the diagnosis and prediction of type 1 diabetes (T1D). They are well established as the biomarkers of the underlying autoimmune pathogenesis (1). Autoantibodies to islet cell antigen, glutamate decarboxylase (GADA), islet antigen-2 (IA-2A), insulin (IAA), and zinc transporter 8 (ZnT8A) are the most commonly used islet autoantibodies at diagnosis (2). As detectable islet autoantibodies overlap between health and disease, a test is usually considered positive for a given islet autoantibody when the antibody level is higher than a 97.5 to 99th percentile of a control population (3, 4). In routine clinical practice, quantitative islet autoantibody results are usually interpreted as positive or negative, and the level of the islet autoantibody is not thought to be clinically meaningful.

Islet autoantibody levels may provide additional information over positivity in T1D at diagnosis. Similar to T1D, autoantibodies to a specific antigen are commonly used for diagnosis in many other autoimmune diseases (such as thyrotropin receptor autoantibodies in Graves disease and tissue transglutaminase in celiac disease). For Graves disease and celiac disease, along with autoantibody positivity for these antigens, autoantibody level at diagnosis is associated with disease severity, prognosis, and treatment success (5, 6). Multiple studies have shown a role for islet autoantibody level in the prediction of onset of T1D: Those with higher levels of IA-2A, IAA, and islet cell antigen have an increased risk of developing T1D in at-risk populations (1, 7–9). However, it is not clear if the islet autoantibody level at diagnosis of T1D, in addition to its interpretation as “positive,” is associated with the clinical phenotype similar to other autoimmune diseases.

In this study, we undertook an analysis of GADA, IA-2A, and ZnT8A levels at diagnosis in a large cohort of participants with T1D, assessing the association of islet autoantibody levels on genetic and clinical characteristics at diagnosis in people with T1D.

## Materials and Methods

### Study Cohorts

We recruited 1644 participants with a clinician-assigned diagnosis of T1D (age at diagnosis range, 4-75 years) who were positive for any of GADA, IA-2A, or ZnT8A at diagnosis. These participants were recruited as part of the United Kingdom–wide ADDRESS-2 study. The detailed protocol for the study has been published previously (10). The included participants were recruited at diagnosis (< 6 months), were older than age 4 years, and insulin-treated from diagnosis. DNA and serum samples, clinical data (including the characteristics of diabetes at diagnosis) were collected at recruitment. The overall cohort characteristics are provided in Supplementary Table S1 and distribution of age at diabetes diagnosis is provided in Supplementary Fig. S1A (11).

We used a second independent replication cohort of 449 participants with clinically diagnosed T1D (age at diagnosis range, 17-81 years) and positivity to any of the 3 islet autoantibodies (GADA, IA-2A, and ZnT8A). The participants were part of the United Kingdom–wide StartRight study (12). All were recruited less than 12 months from diagnosis and insulin-treated from diagnosis. They had random nonfasting serum C-peptide at baseline. They also had a postmeal urine sample for urinary C-peptide/creatinine ratio (UCPCR) at baseline, 1 year, and 2 years from the diabetes diagnosis. The overall cohort characteristics is provided in Supplementary Table S1 and distribution of age at diabetes diagnosis is provided in Supplementary Fig. S1B (11).

### Islet Autoantibody Measurement

#### ADDRESS-2 study

The islet autoantibodies (GADA, IA-2A, ZnT8RA, and ZnT8WA) were measured using established radiobinding assays (RBA) by the Diabetes and Metabolism Group at the University of Bristol, UK, (13, 14) at a median of 11 weeks’ diabetes duration. Results for GADA and IA-2A are expressed in digestive and kidney units/mL (DK units/mL) or arbitrary units (AU/mL) for ZnT8A (the ZnT8A level represents the highest value of either ZnT8RA or ZnT8WA) calculated from standard curves consisting of diluted patient serum samples in antibody-negative serum samples from healthy donors. Positive thresholds were set at the 97.5th percentile of 974 control samples for GADA (≥ 33 DK U/mL), the 98th percentile of 500 control samples for IA-2A (≥ 1.4 DK U/mL), and the 97.5th percentile of 523 healthy school children for ZnT8A (≥ 1.8 AU/mL) (15). The laboratory participates in the Islet Autoantibody Standardization Program (IASP) (Supplementary Table S2) (11).

#### StartRight study

Enzyme-linked immunosorbent assays (ELISAs) (RSR Limited) were used to measure GAD (RRID: AB_2910239, https://scicrunch.org/resolver/AB_2910239), IA-2 (RRID: AB_2910240, https://scicrunch.org/resolver/AB_2910240), and ZnT8 (RRID: AB_2910241, https://scicrunch.org/resolver/AB_2910241) islet autoantibodies on a Dynex DS2 automated ELISA system (Launch Diagnostics) at a median of 15 weeks’ diabetes duration by the Academic Department of Blood Sciences, Royal Devon and Exeter Hospital, UK (16). Positive thresholds were set at the 97.5th percentile of 1559 nondiabetic control individuals (GAD ≥ 11 World Health Organization [WHO] U/mL, IA-2 ≥ 7.5 WHO U/mL, ZnT8 age ≥ 30 years ≥ 10 U/mL, ZnT8 age < 30 years ≥ 65 U/mL). Upper reporting limits for GADA, IA-2A, and ZnT8A were 2000 WHO U/mL, 4000 WHO U/mL, and 2000 U/mL, respectively. The laboratory also participates in IASP (Supplementary Table S2) (11, 17). The analysis of samples from the 2018 IASP workshop showed that islet autoantibodies levels measured by this assay were highly correlated to the RBA used in the ADDRESS-2 cohort for all 3 islet autoantibodies (Supplementary Fig. S2) (11).

### Type 1 Diabetes Genetic Risk Score and Human Leukocyte Antigen Genotypes

We generated weighted Type 1 Diabetes Genetic Risk Score (T1D-GRS) from 30 common T1D genetic variants (single-nucleotide variations, formerly single-nucleotide polymorphisms) for human leukocyte antigen (HLA) and non-HLA loci as described in our previous paper (18). *HLA-DR3-DQ2* and *HLA-DR4-DQ8* were imputed from 2 single-nucleotide variations as described in Barker et al and our previous paper (18, 19).

### Statistical Analysis

We used histograms to assess the distribution of islet autoantibody levels in those positive for that autoantibody. Autoantibody levels with bimodal distributions were split into high- or low-level categories using the nadir (lowest point between distributions). We also used a normal mixture model analysis and likelihood ratio tests to assess whether unimodal, bimodal, or multimodal distributions were best supported by the data. This analysis was performed on log-transformed autoantibody level data so that it was better approximated by a mixture of continuous, symmetric normal distributions. This was implemented using the mixtools package in R (20). We performed Mann-Whitney tests to compare the continuous variables and Pearson chi-square tests were used to compare categorical variables between autoantibody level categories.

For modeling annual UCPCR, the intercept and slopes were determined using mixed-effect models as described previously (21), with random effects at the individual level to allow each individual to contribute multiple C-peptide values at different time points. The benefit of this random-intercept, random-slope model is that it allows for variability between individuals in terms of both C-peptide level at diagnosis (the intercept) and in the percentage change in C-peptide over time (the slope). Groups categorized by GADA and IA-2A level were separately assessed using an interaction term within the mixed-effects model. Due to the slope being on a log scale, they were interpreted in terms of the percentage change per year (calculated from the exponential of the β-coefficient-1). The variability of individual slopes in the longitudinal models was determined using the SD range (calculated by back-transforming the β-coefficient ±1 SD of the slope). All statistical analyses were carried out using Stata/SE 16.0 (StataCorp) unless otherwise stated.

## Results

### Glutamate Decarboxylase and Islet Antigen-2 but not Zinc Transporter 8 Autoantibody Levels Exhibit a Bimodal Distribution at Diagnosis in Type 1 Diabetes

**Figure 1.**
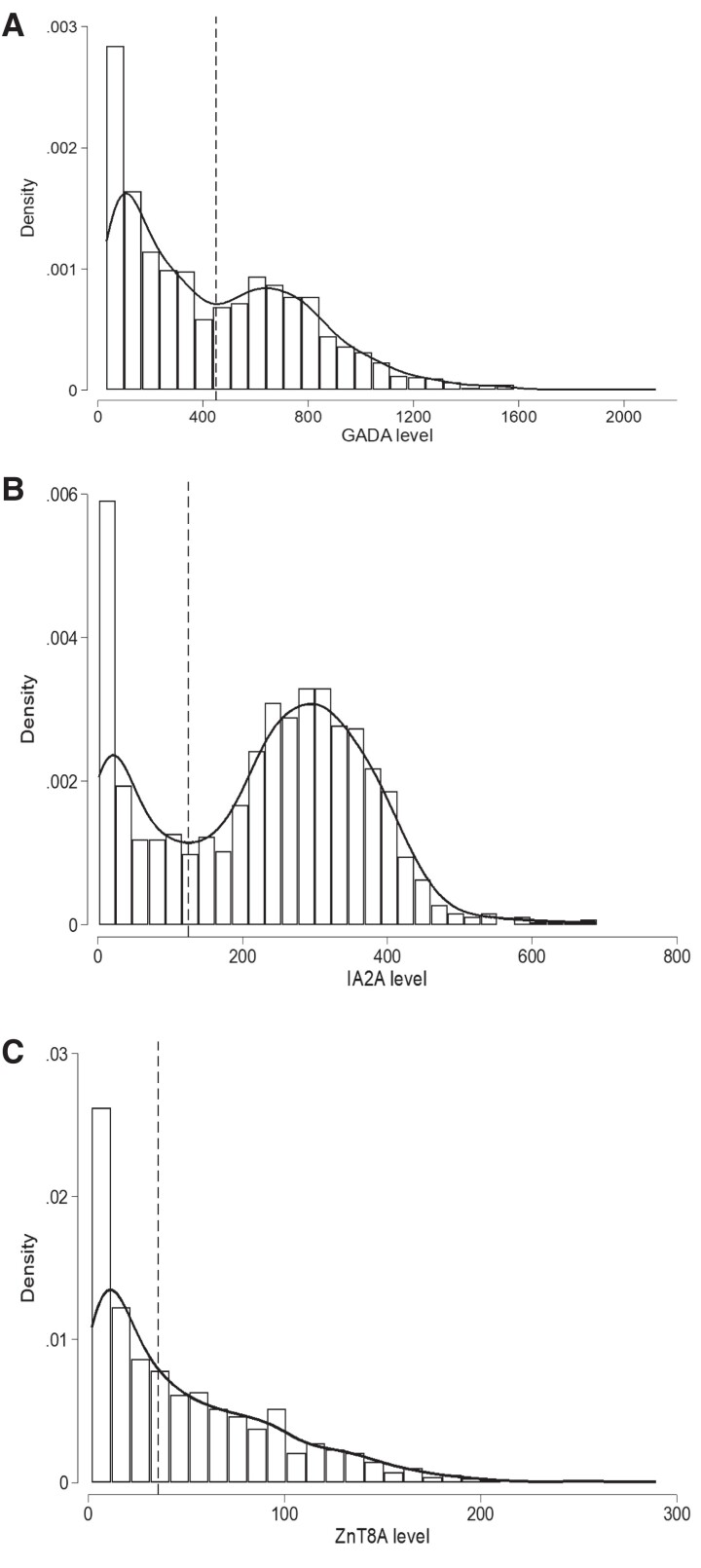
Histograms with kernel density curves showing the distribution of islet autoantibody levels in patients with type 1 diabetes (T1D) at diagnosis. A, Histogram of glutamate decarboxylase (GADA) level at diagnosis measured using radiobinding assay (RBA) for T1D cases that were positive for GADA (n = 1364). GADA level exhibits a bimodal distribution. The nadir value of 452 DK U/mL between the 2 modes is highlighted with a black dashed line and used to defined the high-level GADA group (≥450 DK U/mL) and low-level group (< 450 DK U/mL). B, Histogram of IA-2A level at diagnosis measured using RBA for T1D cases that were positive for islet antigen-2 (IA-2A) (n = 1099). IA-2A level exhibits a bimodal distribution. The nadir value of 125 DK U/mL between the 2 modes is highlighted with a black dashed line and used to defined high IA-2A level group (≥ 125 DK U/mL) and low level group (< 125 DK U/mL). C, Histogram of zinc transporter 8 (ZnT8A) levels at diagnosis measured using RBA for T1D cases that were positive for ZnT8 (n = 954) show a right-skewed distribution. Median value of the distribution (35.6 AU/mL) is highlighted with black dashed lines and used to define high-level ZnT8A (≥ 35.6 AU/mL) and low-level (< 35.6 AU/mL) groups.

We first assessed the distribution of GADA level in GADA-positive T1D individuals (n = 1364), IA-2A level in IA-2A-positive T1D individuals (n = 1099), and ZnT8A level in ZnT8A-positive T1D individuals (n = 954). The distribution of the GADA and IA-2A levels showed 2 peaks consistent with a bimodal distribution (Fig. 1A and B). ZnT8A level showed a single peak with right-skewed distribution (Fig. 1C). The bimodality of GADA and IA-2A levels was also confirmed using a mixture model analysis (Supplementary Fig. S3A and S3B) (11). Specifically, we analyzed IA-2A and GADA levels on the log scale and used a likelihood ratio test (LRT) on 3 degrees of freedom to compare the log-likelihood of a 1-component normal distribution with 2 parameters (1 mean and 1 variance) vs that of a 2-component, 5-parameter normal mixture (2 means, 2 variances, and a weight determining the relative proportion of each component). These analyses yielded overwhelming evidence in favor of the 2-component model (LRT_IA-2A_ = 1219; *P* < 5 × 10^−264^, LRT_GADA_ = 352; *P* < 5 × 10^−76^). For the subsequent analysis, we used the nadir value between the 2 peaks to divide the bimodal distribution of the autoantibody levels into 2 groups (low vs high levels) (see Fig. 1). The nadir value for GADA levels was 450 DK U/mL. All participants with a GADA level lower than this value were grouped into a low-level GADA group (mean level 180, SD ±118, 760/1364 [56%]) and participants with a GADA level above or equal to this value were grouped into a high-level GADA group (770, ±245, 604/1364 [44%]). Similarly, the nadir value of 125 DK U/mL between the 2 peaks of IA-2A levels divided individuals into a low-level IA-2A group (mean level 38, SD ± 35, 296/1099 [27%]) and a high-level IA-2A group (299, ±89, 803/1099 [73%]). The bimodality of GADA level remained after excluding individuals with autoimmune thyroid disease, which is reported to be associated with higher GADA level (Supplementary Fig. S4) (11, 22).

### Higher Glutamate Decarboxylase Levels Were Associated With Later Age At Diagnosis of Type 1 Diabetes, Female Sex, and *HLA-DR3-DQ2*

**Figure 2.**
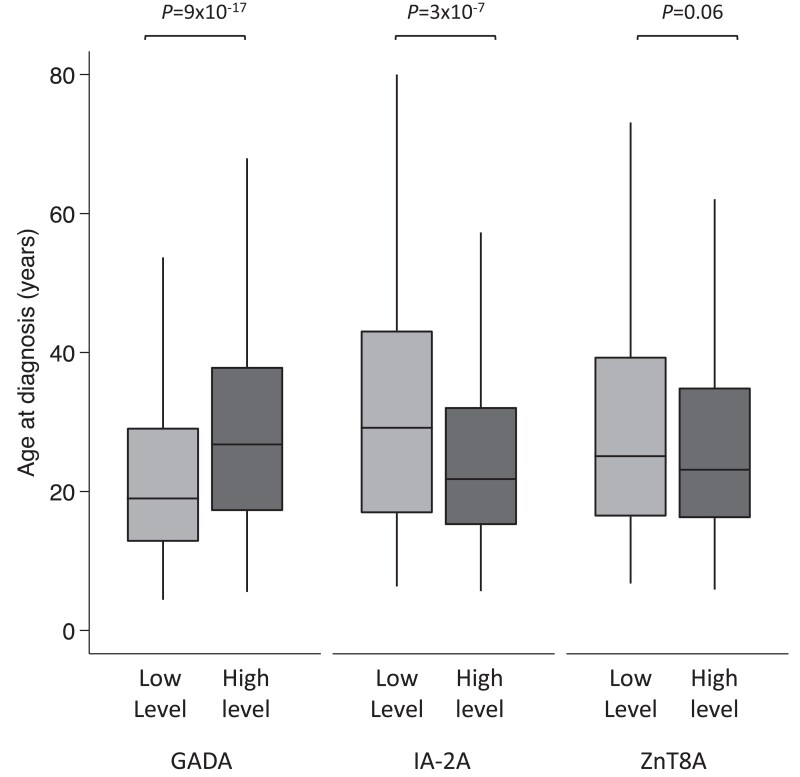
Box plot showing age of diagnosis of type 1 diabetes (T1D) in high- and low-level groups for glutamate decarboxylase (GADA), islet antigen-2 (IA-2A), and zinc transporter 8 (ZnT8A). The nadir value between the 2 modes of GADA level (450 DK U/mL) and IA-2A level (125 DK U/mL) distribution at diagnosis of T1D patients who were positive for respective autoantibodies was used to define high- and low-level categories. There were 604 of 1364 and 760 of 1364 cases in low- and high-level GADA groups and 803 of 1099 and 296 of 1099 cases in high- and low-level IA-2A groups. The median value of ZnT8A level (35.6 AU/mL) was used for defining low- and high-level groups (n = 477 each). Median age of diagnosis was higher for the high-level GADA group (*P* = 9 × 10^−17^), lower for the higher-level IA-2A (*P* = 3 × 10^−7^), and similar between ZnT8A level categories (*P* = .06).

To assess the association of bimodal GADA levels to clinical features at diagnosis, we compared the clinical features between individuals with low-level (lower mode) and high-level (higher mode) GADA as defined earlier (Table 1). Those in the high-level GADA group were diagnosed later compared to the low-level GADA group (median 27 years [interquartile range; IQR, 17-38 years] vs 19 years [IQR, 13-29 years]; *P* = 9 × 10^−17^) (Fig. 2) (see Table 1). They were more likely to be female (52% vs 37%; *P* = 1 × 10^−8^), have a parent with diabetes (20% vs 14%; *P* = .002), and have other autoimmune diseases (13% vs 6%, *P* = 3 × 10^−6^) compared to the low-level GADA group (see Table 1). They had modest enrichment for *HLA-DR3-DQ2* (58% vs 51%; *P* = .006) but had similar T1D-GRS (median 0.273 [IQR, 0.256-0.292] vs 0.275 [IQR, 0.255-0.292]; *P* = .48) based on 30 T1D-associated common variants (23). The presentation characteristics (diabetic ketoacidosis [DKA], weight loss, polyuria, glycated hemoglobin A_1c_ [HbA_1c_], and body mass index [BMI]), the number of other islet autoantibodies, and other islet autoantibody levels were similar between the 2 GADA level groups. In line with this result, more people with adult-onset T1D were in the high-level GADA group compared to childhood-onset T1D (53% vs 33%; *P* = 1 × 10^−13^) (Supplementary Table S4 and Supplementary Fig. S5A and S5B) (11).

**Table 1.** Comparison of clinical characteristics at diagnosis between high and low glutamate decarboxylase (GADA) level groups for GADA-positive type 1 diabetes cases

Characteristic	High-level GADA	Low-level GADA	High vs low
			*P*
**n, % of GADA positives**	604 (44)	760 (56)	
**GADA level, DK U/mL**	723 (594-877)	149 (76-274)	
**Female, %**	317 (52)	282 (37)	1 × 10^−8^*^a^*
**Non-European descent, %**	45 (7)	51 (7)	.60
**Age at diagnosis, y**	27 (17-38)	19 (13-29)	9 × 10^−17^*^a^*
**Duration of diabetes, wk**	10 (6-17)	11 (6-18)	.29
**Hospital admission, %**	434 (72)	577 (76)	.10
**DKA, %**	250 (42)	323 (43)	.74
**Polyuria (%)**	580 (97)	712 (95)	.21
**Weight loss, %**	522 (88)	641 (86)	.23
**HbA_1c_, mmol/mol**	87 (64-110)	81 (58-107)	.01
**BMI**	23.7 (21.4-26.7)	23.2 (20.9-26.2)	.04
**Parent with diabetes, %**	121 (20)	104 (14)	.002*^a^*
**Other autoimmune condition, %**	77 (13)	42 (6)	3 × 10^−6^*^a^*
**T1D-GRS**	0.273 (0.256-0.292)	0.275 (0.255-0.292)	.48
**HLA-DR3-DQ2, %**	353 (58)	387 (51)	.006
**HLA-DR4-DQ8, %**	291 (48)	401 (53)	.09
**No. of positive autoantibodies**			.45
**1, %**	165 (27)	195 (26)	
**2, %**	183 (30)	217 (29)	
**3, %**	256 (42)	348 (46)	
**IA-2A, %**	372 (62)	484 (64)	.43
**IA-2A level, DK U/mL**	254 (91-340)	255 (124-338)	.66
**High-level IA-2A, %**	265 (44)	362 (48)	.17
**ZnT8A (%)**	323 (53)	429 (56)	.27
**ZnT8A level, AU/mL**	43 (13-83)	36 (12-76)	.13
**High-level ZnT8A, %**	180 (30)	215 (28)	.54

Bimodal GADA level distribution was divided into 2 groups using the nadir between the 2 modes at 450 DK U/mL. Values expressed as median (interquartile range) unless stated. Autoantibody levels were assessed in those people who were positive for that antibody.

Abbreviations: BMI, body mass index; DKA, diabetic ketoacidosis; GADA, glutamate decarboxylase; HbA_1c_, glycated hemoglobin A_1c_; HLA, human leukocyte antigen; IA-2A, islet antigen-2; T1D-GRS, Type 1 Diabetes Genetic Risk Score; ZnT8A, zinc transporter 8.

a *P* value lower than threshold the *P* value for multiple comparisons (.05/22 = .0023).

### Higher Islet Antigen-2 Autoantibody Levels Were Associated With Earlier Age at Diagnosis of Type 1 Diabetes and *HLA-DR4-DQ8* and Zinc Transporter 8 Autoantibody Positivity

We next compared the clinical features of low- and high-level IA-2A groups to assess the association of bimodal IA-2A level distribution to clinical features at diagnosis. (Table 2). Contrary to GADA, those in the higher level IA-2A group were diagnosed earlier compared to the lower-level IA-2A group (median 17 years [IQR, 12-25 years] vs 23 years [IQR, 13-34 years]; *P* = 3 × 10^−7^) (see Fig. 2) (see Table 2). They were more likely to be multiple autoantibody positive (60% vs 42% with 3 autoantibodies; *P* = 9 × 10^−9^), positive for ZnT8A (77% vs 52%, *P* = 1 × 10^−15^), and more likely to have higher ZnT8A levels (median level 44 AU/mL [IQR, 16-84 AU/mL] vs 26 [7-54 AU/mL]; *P* = 3 × 10^−6^). Those with higher IA-2A levels were more likely to have *HLA-DR4-DQ8* (66% vs 50%; *P* = 1 × 10^−6^). The presentation characteristics were similar between IA-2A level groups (sex, DKA, weight loss, polyuria, HbA_1c_, and BMI, parent with diabetes) (see Table 2). In line with this result, more people with childhood-onset T1D were in the high-level IA-2A group compared to adult-onset T1D (79% vs 66%; *P* = 2 × 10^−6^) (Supplementary Table S4 and Supplementary Fig. S5C and S5D) (11).

**Table 2.** Comparison of clinical characteristics at diagnosis between high and low islet antigen-2 (IA-2A) level groups for positive IA-2A type 1 diabetes cases

Characteristic	High-level IA-2A	Low-level IA-2A	*P*
**n, % of IA-2A positives**	**803 (73)**	**296 (27)**	
**IA-2A level, DK U/mL**	**295 (237-359)**	**24 (8-65)**	
**Female, %**	**355 (44)**	**117 (40)**	**.16**
**Non-European descent, %**	**48 (6)**	**17 (6)**	**.88**
**Age at diagnosis, y**	**17 (12-25)**	**23 (13-34)**	**3 × 10^−7^*^a^***
**Duration of diabetes, wk**	**12 (7-18)**	**11 (6-18)**	**.20**
**Hospital admission, %**	**650 (81)**	**217 (74)**	**.01**
**DKA, %**	**350 (44)**	**131 (45)**	**.88**
**Polyuria, %**	**767 (97)**	**279 (96)**	**.21**
**Weight loss, %**	**679 (87)**	**244 (86)**	**.20**
**HbA_1c_, mmol/mol**	**76 (56-101)**	**83 (60-114)**	**.01**
**BMI**	**23.2 (21.2-25.9)**	**23.7 (21.5-26.6)**	**.07**
**Parent with diabetes, %**	**110 (14)**	**47 (16)**	**.37**
**Other autoimmune condition, %**	**45 (6)**	**24 (8)**	**.13**
**T1D-GRS**	**0.275 (0.257-0.293)**	**0.275 (0.254-0.294)**	**.73**
**HLA-DR3-DQ2 (%)**	**354 (44)**	**165 (56)**	**6 × 10^−4^*^a^***
**HLA-DR4-DQ8 (%)**	**533 (66)**	**149 (50)**	**1 × 10^−6^*^a^***
**No. of positive autoantibodies**			**9 × 10^−9^*^a^***
**1, %**	**40 (5)**	**38 (13)**	
**2, %**	**283 (35)**	**134 (45)**	
**3, %**	**480 (60)**	**124 (42)**	
**GADA, %**	**627 (78)**	**229 (77)**	**.80**
**GADA level, DK U/mL**	**323 (117-698)**	**374 (149-671)**	**.19**
**High-level GADA, %**	**265 (33)**	**107 (36)**	**.33**
**ZnT8A, %**	**616 (77)**	**153 (52)**	**1 × 10^−15^*^a^***
**ZnT8A level, AU/mL**	**44 (16-84)**	**26 (7-54)**	**3 × 10^−6^*^a^***
**High level ZnT8A, %**	**348 (43)**	**60 (20)**	**2 × 10^−12^*^a^***

Bimodal IA-2A level distribution was divided into low and high level groups using the nadir between the mode at 130 DK U/mL. Autoantibody levels were assessed in those people who were positive for that antibody.

Abbreviations: BMI, body mass index; DKA, diabetic ketoacidosis; GADA, glutamate decarboxylase; HbA_1c_, glycated hemoglobin A_1c_; HLA, human leukocyte antigen; IA-2A, islet antigen-2; T1D-GRS, Type 1 Diabetes Genetic Risk Score; ZnT8A, zinc transporter 8.

a *P* value lower than threshold the *P* value for multiple comparisons (.05/22 = .0023).

### Zinc Transporter 8 Autoantibody Level at Diagnosis Was Not Associated With Age at Diagnosis of Type 1 Diabetes

To assess the association of ZnT8A level to clinical features at diagnosis, we divided 954 T1D patients who were positive for ZnT8A by the median value of the distribution (35.6 AU/mL) due to lack of clear bimodal distribution. No statistically or clinically significant relationship was found between ZnT8A level and age at diagnosis (high level: 17.0 years [IQR, 12-26 years] vs low level: 19 years [IQR, 12-30 years]; *P* = .06) (see Fig. 2) or *HLA-DR3-DQ2* or *DR4-DQ8*. Both groups also exhibited similar presentation characteristics (DKA, weight loss, polyuria, HbA_1c_, and BMI). However, those with higher-level ZnT8A were more likely to be multiple autoantibody positive (71% vs 56% with 3 autoantibodies; *P* = 5 × 10^−6^) and more likely to be positive for IA-2A (86% vs 76%; *P* = 1 × 10^−4^) at higher levels (median level 290 DK U/mL [IQR, 215-359 DK U/mL] vs 245 DK U/mL [IQR, 117-326 DK U/mL]; *P* = 1 × 10^−5^) (Supplementary Table S6) (11). Similar results for the lack of association of ZnT8A level to age at diagnosis were observed with linear regression analysis of log ZnT8A level to age at diagnosis (β = −0.55, 95% CI, −1.2 to 0.08; *P* = .09).

### Glutamate Decarboxylase Autoantibody–Negative Individuals Were Younger at Diagnosis in Comparison to Islet Antigen-2 Autoantibody/Zinc Transporter 8 Autoantibody–Negative Individuals Who Were Older at Diagnosis

The comparison of people with low-level GADA to negative GADA (positive for IA-2A and/or ZnT8A) showed that the GADA-negative individuals were diagnosed younger (median 14 years [IQR, 10-21 years] vs 19 years [IQR, 13-29 years]; *P* = 2 × 10^−12^) and had higher DR4-DQ8 (53% vs 69%; *P* = 6 × 10^−6^) (Supplementary Table S3) (11). In contrast, individuals negative for IA-2A (positive for GADA and/or ZnT8A) were diagnosed older compared to low-level IA-2A individuals (median 28 years [IQR, 18-38 years] vs 23 years [IQR, 13-34 years]; *P* = 6 × 10^−6^) (Supplementary Table S5) (11). Similar results were also observed for ZnT8A (median 25 years [IQR, 15-37 years] for those who were ZnT8A negative vs 19 years [IQR, 12-30 years] with low-level ZnT8A; *P* = 6 × 10^−10^) (Supplementary Table S6) (11). Both IA-2A– and ZnT8A-negative groups were highly enriched for GADA-positive individuals (93% and 88%, respectively).

### Bimodal Distributions of Glutamate Decarboxylase and Islet Antigen-2 Autoantibody Levels Were Also Observed in a Second Independent Cohort

To replicate our results with a different assay and different cohort, we analyzed GADA, IA-2A, and ZnT8A levels in 449 patients with T1D at diagnosis from the StartRight study, in which islet autoantibody levels were measured using ELISA assays, another commonly used assay for islet autoantibody measurement.

Similar to our primary cohort, GADA and IA-2A levels showed a bimodal distribution in this replication cohort, with ZnT8A showing one peak with right-skewed distribution (Supplementary Fig. S6) (11). The shape of the distribution is different in this cohort due to the clinical laboratory conducting the ELISAs not reporting results that are outside the standard curve leading to truncation at higher levels.

Using the same method as our primary cohort, we divided the GADA and IA-2A bimodal distributions into high- and low-autoantibody level groups using the nadir between the peaks (see Supplementary Fig. S6) (11). Similarly to our primary analysis, those in the high-level GADA group were diagnosed later compared to the low-level GADA group (40 years [IQR, 31-53 years] vs 30 years [IQR, 24-38 years]; *P* = 6 × 10^−13^) and were more likely to be female (60% vs 43%; *P* = 5 × 10^−4^). There were no differences in presentation characteristics, parent with diabetes, or random nonfasting C-peptide at diagnosis (Supplementary Table S7) (11). However, people with high-level GADA showed a trend toward faster decline of C-peptide in the first 2 years compared to those with a low level of GADA (annual decline in UCPCR −48% [95% CI, −41% to −55] vs −42% [95% CI, −33% to −50%]; *P* = .258) (Supplementary Fig. S8A) (11). The participants with higher IA-2A levels were younger (median age, 28 years [IQR, 22-48 years] vs 33 years [IQR, 25-46 years]) compared to the ones with lower IA-2A levels, as our primary cohort, but this difference was not statistically significant (Supplementary Table S8) (11). The baseline and annual decline in C-peptide was also similar between the 2 IA-2A level groups (−50%, 95% CI, −31% to −63%; vs −46%, 95% CI, −38% to −54%; *P* = .708) (Supplementary Fig. S8B) (11).

## Discussion

Our study shows that GADA and IA-2A level at diagnosis of T1D show clear bimodal distributions. Dichotomizing levels into high or low groups according to the observed modes exhibits strong associations with age at T1D diagnosis but not with severity of T1D at diagnosis.

The bimodality of GADA and IA-2A is a novel finding in T1D and may point toward a T1D pathogenesis. There have been multiple studies of islet autoantibody levels in at-risk populations for T1D (1, 7–9) and within T1D populations but none of the studies to our knowledge have reported that GADA and IA-2A levels have a bimodal distribution. However, the bimodality of GADA levels but not IA-2A levels has been reported in people with latent autoimmune diabetes (LADA) (24, 25). Older-onset T1D patients have a higher proportion of GADA whereas childhood-onset diabetes patients have a higher proportion of IA-2A, IAA, and ZnT8A (15). We found that levels of GADA and IA-2A also follow the same pattern with higher GADA levels in older individuals and higher IA-2A levels in younger individuals. This does not confirm but may suggest that a higher level of autoantibody in an individual points toward the triggering (first) autoantibody and a lower level of autoantibody in an individual points toward spreading autoantibodies. This can also be supported by the observation that those positive for *HLA-DR3-DQ2* had higher levels of the associated triggering GADA and lower levels of spreading IA-2A, and the reverse was observed in those positive for *HLA-DR4-DQ8* (higher levels of the associated triggering IA-2A but lower GADA levels) (Supplementary Fig. S7) (11). ZnT8A levels were not seen to differ between positivity for either *HLA-DR4-DQ8* or *HLA-DR3-DQ2*.

The additional factors including genetic predisposition underlies the observed bimodality. There was an enrichment of *HLA DR3-DQ2* and *HLA DR4-DQ8* in people with higher GADA levels and IA-2A levels, respectively. Both these associations are well described with the positivity of the respective autoantibodies but not with the autoantibody levels (26, 27). The difference in HLA susceptibility suggests a role for humoral immunity and antigen recognition as one of the factors underlying bimodality. However, the association with HLA was modest in our study and the overall genetic risk score was similar with high- and low-level autoantibody groups, suggesting that there are additional factors that are responsible for the observed bimodality. Previous studies have shown that high GADA levels are correlated with higher affinity autoantibodies, the central and C-terminal epitopes, and multiple autoantibodies positivity (28). We did not observe the association of high-level GADA with multiple autoantibodies, but we did observe the association of higher IA-2A level with multiple autoantibodies. These data suggest that affinity, the difference in epitopes, and the presence of other autoantibodies may also contribute toward bimodality.

GADA and IA-2A levels are associated with age at onset but in opposite directions. The participants who had high GADA levels were nearly 7 years older at diagnosis compared to the ones with low-level GADA. This was replicated in a second independent cohort of adults with T1D. Contrary to GADA, participants with high IA-2A levels were 5.4 years younger at diagnosis compared to those with low IA-2A levels. We did not observe a statistically significant reduction in age at diagnosis in people with the high level of IA-2A in our second independent cohort. This may be due to the combination of the overall older age of onset of diabetes (median, 34 years), longer time to islet autoantibody measurement from the diagnosis (25% were measured at > 31 weeks), and use of ELISA compared to our primary cohort. Interestingly, we note that both GADA and IA-2A levels but not ZnT8A level follow the same association with age as positivity of these autoantibodies (15, 29, 30). In contrast to our findings in T1D, studies of LADA have shown that higher GADA level is associated with early age of onset. This may relate to the very different populations studied and the relationship between age and prior prevalence of autoimmune diabetes that, as recently suggested, may markedly alter antibody false-positive rates in populations of apparent type 2 diabetes (25, 30, 31).

The observed association of high level of GADA with concurrent other autoimmune disease was mainly due to thyroid/celiac autoimmunity (68/77 in high-level GADA and 34/42 in low-level GADA). This association is likely due to the shared HLA risk alleles DR3-DQ8 for thyroid and celiac autoimmunity and high level of GADA in our study (32, 33).

Autoantibody level at diagnosis was not strongly associated with severity of presentation of T1D but may be associated with β-cell function at follow-up. We did not observe an association of autoantibody levels with symptoms of hyperglycemia, BMI, HBA_1_c, and C-peptide at diagnosis. These results contrast with previous studies of LADA in which high levels of GADA were associated with lower BMI, C-peptide, and higher HbA_1c_ (25). Interestingly, we did observe a trend toward a lower C-peptide at follow-up in our second cohort. The small sample size and shorter follow-up means this finding was not statistically significant. A similar trend has been reported with GADA level in a recent cross-sectional study of patients with T1D (34). This observed association if replicated in the larger cohort provides an exciting opportunity to identify T1D subtypes, which has important clinical significance for the prediction of islet function and early intervention to prevent severe metabolic complications. Our findings have important implications for the prediction, treatment, and prognosis of T1D. It is well known that T1D is a heterogeneous disease with heterogeneity in islet autoantibodies, β-cell function, genetics, as well as response to immunomodulatory therapy (35). The research to date has focused mainly on the positivity of autoantibodies rather than autoantibody levels in T1D. We believe that autoantibody levels showing a bimodality for GADA and IA-2A is an important consideration in understanding the heterogeneity and pathogenesis of T1D. It is well known that immunomodulatory therapy has a variable response in β-cell function in clinical trials (36). Currently, the reason for this variable response is not entirely known but is proposed to be due to variation in T-cell response (37, 38). The bimodality of the levels may represent a surrogate marker of a specific immune response and identify the subgroup of individuals with a differential response to immunomodulatory therapy, but this needs testing in further studies. This, along with the association of GADA level with C-peptide in a recent study, provides an exciting possibility of a stratified approach to T1D treatment and prognosis that is currently lacking (34). Our findings also make a strong case to assess the usefulness of the bimodality of GADA and IA-2A levels in the prediction models of progression of diabetes in at-risk population in addition to autoantibody positivity.

Our islet autoantibody levels were assessed using RBA in our primary cohort. We validated our findings using a replication cohort and a second method of islet autoantibody assessment (ELISA) that is more commonly used in routine clinical laboratories. However, both assays showed a high level of correlation for all 3 autoantibodies in the same samples during the IASP 2018 workshop (see Supplementary Fig. S2) (11). This was also in line with previous studies (39, 40). This suggests that our findings are applicable to levels measured by both methods. Although a bimodal distribution of GADA and IA-2A was observed using both assay methods, the shape of the distributions does look different between assays. We believe this is due to the clinical laboratory conducting the ELISAs not reporting results that are outside the standard curve causing truncation at both ends in comparison with the RBA laboratory, which reports extrapolated results.

Our study was limited by the use of 97.5th to 98th percentiles of the controls to define autoantibody positivity in our study. Although this cutoff is widely used in clinical practice, the use of these cutoffs, despite the higher prior probability of our cohort, would have led to the inclusion of a small number of people with low levels as positive for each autoantibody (up to 2.5% for each autoantibody). This may have a small effect on the distribution at the lower levels but is unlikely to change the bimodality and overall conclusion of our study due to the large sample size of our cohort. Also, we only had C-peptide information at diagnosis and follow-up in one of the study cohorts; therefore, we are limited in our ability to assess the effect of autoantibody-level distribution on β-cell function. We did not study IAAs as this would not be appropriate due to the nature of our cohorts being recruited in the weeks after diagnosis of T1D and commencement of insulin therapy.

In conclusion, we show that GADA and IA-2A levels exhibit a bimodal distribution at diagnosis of T1D, which is biologically important to the understanding of the heterogeneity of T1D and opens the exciting possibility of further research to assess its implication for the prediction, treatment, and prognosis of T1D.

## Data Availability

Some or all data sets generated during and/or analyzed during the present study are not publicly available but are available from the corresponding author on reasonable request.

## Abbreviations

BMI: body mass index
DKA: diabetic ketoacidosis
ELISA: enzyme-linked immunosorbent assay
GADA: glutamate decarboxylase autoantibody
HbA_1c_: glycated hemoglobin A_1c_
HLA: human leukocyte antigen
IA-2A: islet antigen-2 autoantibody
IAA: insulin autoantibody
IASP: Islet Autoantibody Standardization Program
IQR: interquartile range
LADA: latent autoimmune diabetes
LRT: likelihood ratio test
RBA: radiobinding assay
T1D: type 1 diabetes
T1D-GRS: Type 1 Diabetes Genetic Risk Score
UCPCR: urinary C-peptide/creatinine ratio
WHO: World Health Organization
ZnT8A: zinc transporter 8 autoantibody
